# Distortion-Aware Routing and Parameter-Shared MoE for Multispectral Remote Sensing Super-Resolution

**DOI:** 10.3390/s26072186

**Published:** 2026-04-01

**Authors:** Shuo Yang, Shi Chen, Yuxuan Liu, Tianhui Zhang

**Affiliations:** 1 National Space Science Centre, Chinese Academy of Sciences, Beijing 100190, China; yangshuo23@mails.ucas.ac.cn (S.Y.); liuyuxuan231@mails.ucas.ac.cn (Y.L.); zhangtianhui22@mails.ucas.ac.cn (T.Z.); 2 University of Chinese Academy of Sciences, Beijing 100049, China

**Keywords:** multispectral imaging, super-resolution, mixture-of-experts, parameter-efficient fine-tuning, low-rank adaptation, remote sensing

## Abstract

Multispectral remote sensing image super-resolution (RSISR) aims to reconstruct high-frequency details while preserving cross-band structural consistency under strict computational budgets. However, real-world satellite imagery exhibits heterogeneous distortions, ranging from band-dependent noise to spatially varying texture degradation, rendering uniform restoration strategies suboptimal. To address these challenges, we propose a unified framework that integrates cue extraction, expert specialization, and efficiency-aware restoration. Specifically, a Distortion-Aware Feature Extractor (DAFE) explicitly encodes distortion cues by synthesizing fixed frequency bases, learnable residual components, lightweight spatial edge representations, and noise proxies. Subsequently, a Distortion-Aware Expert Choice (DAEC) router utilizes these cues to establish distortion-conditioned affinities and performs capacity-constrained, load-balanced expert assignment. Finally, a parameter-shared Mixture-of-Experts (PS-MoE) architecture employs shared expert parameters across spectral bands, augmented by band-wise low-rank adapters, to enable coarse-to-fine restoration with minimal computational overhead. Extensive experiments on the SEN2VEN*μ*S and OLI2MSI datasets demonstrate that the proposed method achieves a PSNR of 49.38 dB on SEN2VEN*μ*S 2×, 45.91 dB on SEN2VEN*μ*S 4×, and 45.94 dB on OLI2MSI 3×. Compared to the strongest baseline for each task, our method yields PSNR improvements of 0.12 dB, 0.10 dB, and 0.09 dB, respectively, while simultaneously reducing FLOPs and parameter counts. These results confirm that explicit distortion modeling and parameter-shared expert specialization provide an effective and computationally efficient solution for multispectral remote sensing image super-resolution.

## 1. Introduction

Multispectral remote sensing image super-resolution (RSISR) aims to reconstruct high-resolution imagery from low-resolution multispectral observations while preserving both fine spatial details and cross-band structural consistency. This task is pivotal for a wide range of downstream applications, including land-cover mapping, environmental monitoring, and precision agriculture, where spatial detail directly influences interpretation quality. In contrast to natural image super-resolution, RSISR presents distinct challenges because real satellite observations are compromised by heterogeneous degradations, such as edge blurring, texture attenuation, band-dependent noise, and sensor-specific distortions, which vary across regions and spectral bands. Under these conditions, the application of a uniform restoration strategy to all tokens or bands often proves suboptimal.

Recent Transformer-based restoration models have substantially advanced image super-resolution by improving long-range dependency modeling and texture reconstruction [1,2,3,4]. In a broader context, Transformer-based models have been extensively explored in multispectral and hyperspectral remote sensing for tasks beyond super-resolution, particularly classification. For instance, DFTN introduces dual-frequency modeling to capture complementary high- and low-frequency representations; MSNAT strengthens multiscale neighborhood attention for spatial pattern modeling; and SiT emphasizes scale interaction for spectral–spatial feature learning [5,6,7]. These studies further validate the suitability of Transformer architectures for modeling complex multi-band remote sensing data. However, two limitations remain particularly pertinent to RSISR. First, although existing methods can implicitly learn degradation patterns from data, they often fail to explicitly characterize distortion types or severities. Consequently, the model lacks sufficient guidance to distinguish whether a token is dominated by missing high-frequency detail, structural blur, or noise contamination [8,9,10,11,12]. Second, while sparse Mixture-of-Experts (MoE) architectures provide an attractive mechanism to increase model capacity without a proportional increase in computation [13,14,15,16,17,18], their routing decisions typically rely on generic semantic features rather than restoration-oriented distortion cues. Moreover, in multispectral settings, the naive assignment of separate experts to different bands can result in substantial overhead regarding parameters and memory [19,20].

To address these challenges, we propose a distortion-aware and parameter-efficient framework for RSISR. The core concept involves the explicit extraction of lightweight distortion cues to guide expert routing, followed by adaptive restoration using shared experts and band-specific specialization. Specifically, we first introduce a Distortion-Aware Feature Extractor (DAFE), which encodes frequency and spatial degradation information by combining fixed frequency bases, learnable residual frequency components, edge responses, and noise proxies. These cues provide an explicit description of local degradation characteristics and complement the spatial features of the backbone. Leveraging this fused representation, we then introduce a Distortion-Aware Expert-Choice (DAEC) router, which constructs distortion-conditioned affinities and performs capacity-constrained, load-balanced token assignment. Consequently, different experts can focus on distinct restoration modes while maintaining predictable resource usage. Finally, we develop a Parameter-Shared Mixture-of-Experts (PS-MoE) architecture that shares the main expert parameters across spectral bands and equips them with lightweight band-wise LoRA adapters [19,20]. This design preserves cross-band consistency while retaining sufficient flexibility to model band-specific statistics.

The rationale for explicit distortion modeling is that degradations most relevant to super-resolution are often manifested as high-frequency attenuation and structural discontinuities. Classical signal representations, such as the discrete cosine transform and wavelets [8,9], are well-suited for characterizing these variations. Rather than utilizing frequency features merely as auxiliary descriptors, our method converts them into routing-relevant cues, enabling the model to better distinguish regions requiring different restoration behaviors. This alignment ensures that the routing process corresponds more closely to the actual degradation structure of multispectral imagery. Concurrently, the use of capacity-constrained expert-choice routing provides a practical mechanism to balance restoration quality and computational cost, which is particularly critical for high-resolution multi-band inputs. Furthermore, parameter sharing across bands avoids the redundancy of fully independent per-band experts, while the band-wise LoRA modules preserve lightweight specialization where it is most necessary.

**Figure 1:**
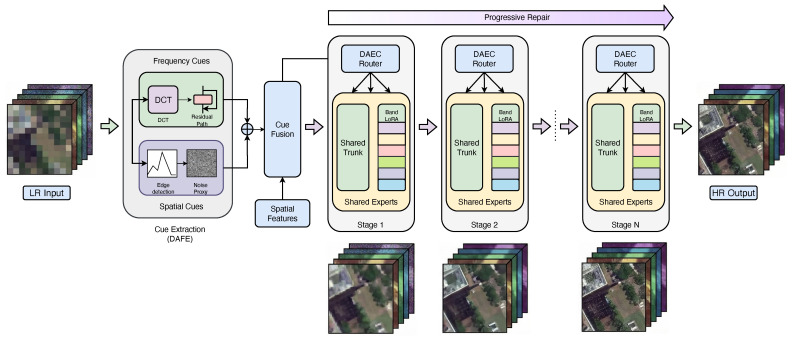
Overall architecture. Our framework follows a cue extraction → expert specialization → progressive repair pipeline. First, the Distortion-Aware Feature Extractor (DAFE) derives lightweight routing cues from complementary frequency and spatial distortion signals, including DCT-based responses, residual frequency cues, edge magnitude, and noise proxies. These cues are fused with backbone spatial features and passed to the Distortion-Aware Expert-Choice (DAEC) router, which performs capacity-aware expert-choice routing. The routed tokens are then processed by stacked PS-MoE blocks, where a shared expert trunk is combined with band-wise LoRA adapters to achieve parameter-efficient multispectral specialization. Repeating DAEC + PS-MoE across depth produces a progressive coarse-to-fine restoration process, finally reconstructing the HR multispectral output.

Figure 1 illustrates the overall architecture of the proposed framework. Let $X\in R^{N\times d}$ denote the tokenized backbone features. DAFE produces distortion-aware cues $F\in R^{N\times k}$, which are fused with *X* to form the routing representation. DAEC then computes expert affinities and performs capacity-aware assignment; subsequently, the selected experts process the routed tokens. The PS-MoE blocks are stacked across depth so that the network progressively restores coarse structures in shallow layers and refines fine textures in deeper layers, finally reconstructing the high-resolution multispectral output.

The primary contributions of this work are summarized as follows. First, we present a unified distortion-aware RSISR framework that explicitly links distortion cue extraction, expert routing, and parameter-efficient restoration. Second, we design DAFE and DAEC to enable routing decisions based on degradation-aware signals rather than generic features, while preserving stable and predictable expert utilization through capacity and load-balancing constraints. Third, we propose a PS-MoE architecture with band-wise LoRA adaptation, which achieves a favorable balance between parameter sharing and spectral specialization. Fourth, extensive experiments in Section 4 validate that the proposed method achieves state-of-the-art performance with competitive memory and computational efficiency.

The remainder of this paper is organized as follows. Section 2 reviews related work on image restoration, sparse expert models, parameter-efficient adaptation, and frequency priors. Section 3 presents the proposed method in detail. Section 4 reports the experimental results and analyses. Section 5 further discusses the interpretation of the results, their relation to existing literature, and the limitations of the proposed method. Section 6 concludes the paper.

## 2. Related Work

### 2.1. Transformer-Based Image Restoration and SR

Transformers have established themselves as the dominant architecture in super-resolution (SR) by exploiting various attention mechanisms. Methods employing windowed attention [1,3,4], such as SwinIR and Swin2SR, introduce hierarchical self-attention into low-level vision tasks. These approaches achieve a favorable balance between reconstruction quality and efficiency, serving as strong baselines for image restoration. Collectively, these studies demonstrate that window-based attention effectively captures local structures while maintaining computational feasibility, which is particularly critical for high-resolution SR. Concurrently, models focusing on global context interactions [2,21,22,23], such as Restormer, highlight the efficacy of efficient attention in modeling long-range dependencies for high-resolution restoration. These works further indicate that incorporating long-range context enhances the recovery of spatially distributed textures and structures that purely local operations fail to handle adequately. Additionally, recent studies have proposed efficient approximations to further optimize performance [24,25,26,27]. These developments build upon foundational Convolutional Neural Network (CNN) benchmarks [28,29,30,31,32] and perceptual generative approaches [33,34,35]. While prior CNN-based methods established robust reconstruction standards through residual learning and dense feature reuse, they typically prioritize texture synthesis. In summary, although both CNN and Transformer-based SR methods significantly improve reconstruction fidelity, most standard architectures continue to employ a uniform processing pathway across the entire image. Consequently, standard architectures often remain agnostic to heterogeneous spatial and spectral distortions. They lack the mechanisms to dynamically allocate computational resources according to the severity of degradation. Our distortion-aware routing addresses this limitation by extracting explicit cues to guide adaptive capacity.

### 2.2. Sparse Mixture-of-Experts and Routing

Sparse Mixture-of-Experts (MoE) models scale capacity by activating only a subset of experts per token [13,14,16]. Seminal studies, including GShard and Switch Transformer, demonstrate that sparse routing effectively scales model parameters while maintaining manageable per-token computation. A key finding from these studies is that conditional computation increases model capacity without incurring the full inference cost associated with dense expert activation. This paradigm has been successfully adapted to computer vision tasks, as exemplified by V-MoE [15], which confirms that conditional expert activation benefits visual representation learning beyond language models. These results imply that expert specialization offers benefits in visual domains, particularly when inputs exhibit diverse patterns that a single shared transformation cannot adequately address. We build upon Expert-Choice (EC) routing [17], a strategy that ensures predictable capacity by allowing experts to select the top-*C* tokens. By improving load balancing and mitigating token dropping under constrained budgets, EC routing provides the stability required for restoration tasks. Therefore, while prior research has established the scalability and stability advantages of sparse expert selection, existing routing signals are typically derived from generic token features rather than explicit degradation descriptors. In contrast to standard EC, which relies on generic features, we compute affinities from explicit distortion tokens (DAFE). This enables experts to specialize in SR-specific degradation modes, such as noise or blur, under strict capacity constraints.

### 2.3. Parameter-Efficient Adaptation

Parameter-Efficient Fine-Tuning (PEFT) methods, including adapters [20] and Low-Rank Adaptation (LoRA) [19], facilitate lightweight specialization. These approaches demonstrate that effective adaptation is achievable with significantly fewer trainable parameters and lower memory costs compared to full fine-tuning. The primary insight is that task-specific adaptation can be achieved while largely preserving the pretrained backbone, making these methods particularly attractive when model scale or deployment costs are constrained. Similarly, techniques such as AdapterFusion [36] support multitasking. Consequently, these methods provide a viable pathway for specializing large backbones without incurring prohibitive parameter growth. Collectively, PEFT studies suggest that modular adaptation enhances flexibility and reusability, effectively avoiding the parameter explosion associated with fully independent branches. In the context of multispectral SR, the naive instantiation of experts for each spectral band leads to parameter explosion. Our Parameter-Shared MoE (PS-MoE) mitigates this issue by sharing expert trunks across bands while attaching band-specific LoRA modules [19,20], thereby preserving spectral specialization with minimal memory overhead.

### 2.4. Frequency Priors and RSISR Challenges

Explicit frequency cues, such as the Discrete Cosine Transform (DCT) [8] and wavelets [9], have long been integrated into neural architectures like MWCNN [10], FcaNet [11], and FreqNet [12]. These studies indicate that frequency decomposition complements purely spatial representations by characterizing structures across different scales and frequency bands. A consistent observation across these methods is that frequency-domain priors enable neural networks to better capture the high-frequency details and multi-scale structures essential for image restoration. However, existing methods typically treat frequency as a passive feature rather than an active control signal. In Remote-Sensing SR (RSISR), challenges such as cross-sensor inconsistencies and atmospheric effects [37,38,39] require methods capable of localizing distortions. Benchmarks such as SEN2VEN*μ*S and OLI2MSI reflect the realistic cross-sensor and cross-band degradations encountered in practical settings. These benchmarks highlight that performance in RSISR depends not only on generic reconstruction capabilities but also on robustness to heterogeneous degradations arising from different sensors, bands, and acquisition conditions. Our DAFE module transforms frequency and spatial cues into routing signals, allowing the model to adapt to the diverse degradation patterns found in these benchmarks [38,39].

## 3. Method

### 3.1. Overview

Our method operationalizes cue extraction → expert specialization → progressive repair with three tightly coupled components—DAFE → DAEC → PS-MoE—as illustrated in Figure 1. The Distortion-Aware Feature Extractor (DAFE) first derives compact routing cues from both frequency-aware and spatial distortion descriptors. These cue features are fused with backbone spatial tokens and fed into the Distortion-Aware Expert-Choice (DAEC) router, which performs capacity-aware expert-choice assignment. The selected experts are instantiated as PS-MoE blocks with a shared trunk and band-wise LoRA adapters, enabling parameter-efficient yet band-sensitive restoration. By stacking DAEC + PS-MoE blocks across depth, the network performs progressive coarse-to-fine repair, where shallow layers focus more on structural recovery and deeper layers refine high-frequency details.

**Figure 2:**
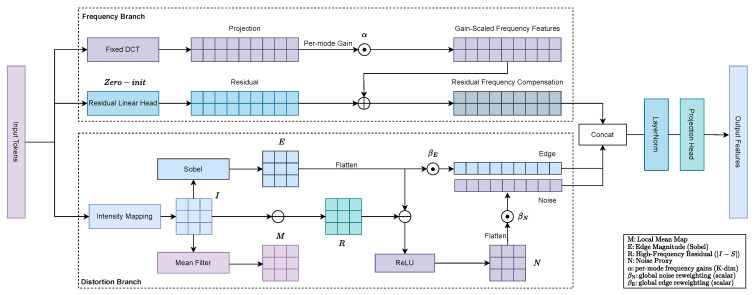
Distortion-Aware Feature Extractor (DAFE). The module produces lightweight routing features from two complementary views. Frequency branch (**top**) projects token features onto a fixed DCT basis with per-mode gains and adds a zero-initialized residual linear head to capture task-specific frequency deviations. Distortion branch (**bottom**) reconstructs a 2D intensity map from tokens, computes the Sobel edge magnitude and a high-frequency residual via a 3×3 mean filter, and obtains a noise proxy using ReLU(residual−edge). The frequency vector and the two scalars (edge, noise) are concatenated, layer-normalized, and projected to produce routing features for MoE.

Let $X\;\in \;R^{N\times d}$ denote the tokenized features from B input bands. The Distortion-Aware Feature Extractor (DAFE) (Figure 2 and Section 3.2) produces distortion cues $F\;\in \;R^{N\times D}$ by projecting tokens onto a fixed DCT basis $B\;\in \;R^{K\times d}$ with per-mode gains $\gamma \;\in \;R^{K}$, adding a zero-initialized residual head, and appending two scalar proxies that summarize edge magnitude and noise.

We fuse the distortion cues with spatial features as

(1) $$\tilde{X}=\left[\mathrm{LN}\left(X\right)\;∥\;\mathrm{LN}\left(F\right)\;W_{f}\right]\in R^{N\times (d+d_{g})},$$

where $d_{g}$ denotes the dimensionality of the projected cue space used by the gate, and $W_{f}\in R^{D\times d_{g}}$ is a learnable projection matrix mapping the cues to the gating dimension. Based on the fused representation, the expert affinities are computed as

(2) $$A=\tilde{X}W_{g}/\tau ,$$

where $W_{g}\in R^{(d+d_{g})\times E}$ is the learnable gating weight matrix, and the temperature τ controls the sharpness of the routing distribution.

**Figure 3:**
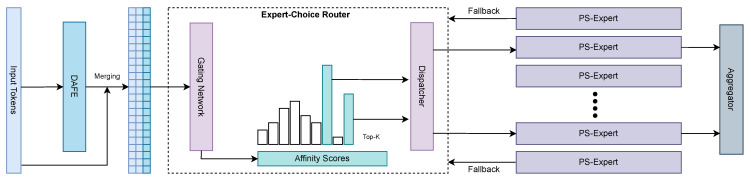
Overview of Distortion-Aware Expert Choice (DAEC). DAFE provides explicit distortion and frequency cues that are fused with token features and fed into a gating network to produce per-expert affinity scores. Each expert then claims the Top-*C* tokens it is most confident in handling (capacity-constrained expert choice), while unclaimed tokens follow a lightweight fallback path. A dispatcher sends claimed tokens to PS-Experts (parameter-shared experts equipped with lightweight band adapters) and the aggregator fuses expert outputs with residual connections. DAEC operationalizes the narrative of the paper: first localize distortions, then repair them progressively in a divide-and-conquer fashion.

The distortion-aware expert-choice (DAEC) router (Figure 3 and Section 3.3) uses expert choice with capacity, where each expert e∈{1,…,E} claims its Top-*C* tokens where C=⌊ϕ·N/E⌋ and ϕ≥1 is a slack factor. The per-token fan-out is limited to ρ∈{1,2} after conflict resolution, yielding predictable memory and latency without token drops. Claimed tokens are dispatched, aggregated with soft weights within the admitted set, and unclaimed tokens follow a lightweight fallback to guarantee coverage. Auxiliary terms $L_{\mathrm{imp}}$, $L_{\mathrm{cap}}$, and $L_{z}$ stabilize utilization and logits during training.

**Figure 4:**
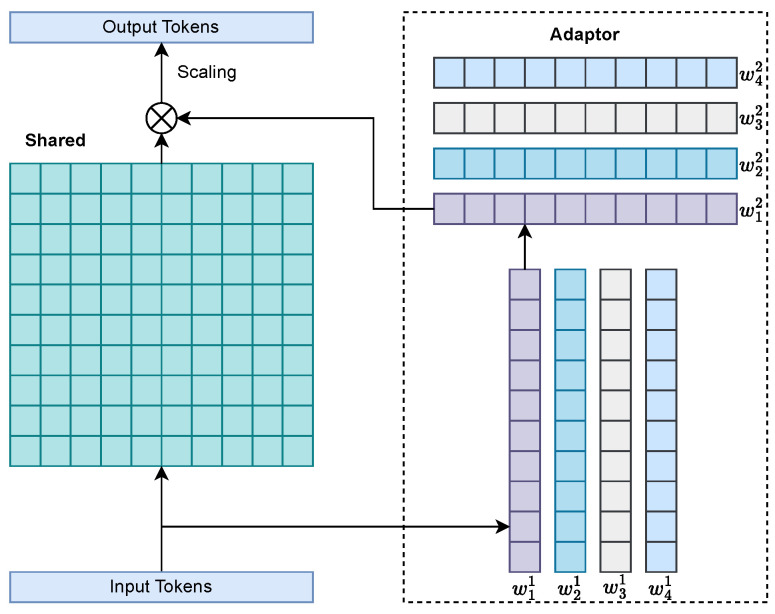
PS-MoE overview. Each expert shares a lightweight two-layer MLP trunk across bands (gray), while per-band LoRA adapters (colored blocks) inject specialization at both layers. The router assigns sparse gates (Expert-Choice). Outputs from active experts are combined by the gated sum. This design approaches per-band capacity with markedly lower parameters and VRAM.

The parameter-shared MoE (PS-MoE) experts (Figure 4 and Section 3.4) share a two-layer MLP trunk across bands while attaching band-wise LoRA adapters of rank *r* at both projections, which preserves cross-band priors yet enables band-specific specialization at negligible inference overhead. Optionally, the small-band embedding $b\;\in \;R^{d_{b}}$ is concatenated to $\tilde{X}$ to make routing band-aware without replicating experts.

Stacking these DAEC + PS-MoE blocks across the depth yields the progressive repair process visualized in Figure 1; shallow blocks mainly recover coarse structures and suppress artifacts, whereas deeper blocks emphasize fine textures and band-dependent high-frequency details.

In summary, DAFE extracts explicit distortion cues, DAEC converts these cues into capacity-aware routing decisions, and PS-MoE performs efficient multispectral restoration through shared experts with band-wise adaptation, together forming a unified and interpretable restoration pipeline.

In summary, DAFE supplies interpretable distortion tokens, DAEC converts them into capacity-aware decisions, and PS-MoE executes repairs efficiently across B bands, forming a unified pipeline that achieves a favorable balance between fidelity and computational efficiency.

### 3.2. DAFE: Distortion-Aware Feature Extractor

Design and role. As shown in Figure 2, DAFE converts token features into interpretable distortion cues that drive routing. It couples a frequency view—fixed DCT modes with learnable per-mode gains and a residual head—with a spatial view that produces compact edge and noise proxies. The result is a low-cost, stable signal that complements spatial features and improves expert specialization.

Setup and notation.

Let $X\;\in \;R^{N\times d}$ denote tokenized features from B input bands, where N=BHW. DAFE outputs the routing features $F\;\in \;R^{N\times D}$.

Frequency branch (fixed priors + learnable residual).

We build a DCT-II basis, $B\;\in \;R^{K\times d}$ (rows $\ell_{2}$-normalized, low→high frequency by row index) and apply learnable per-mode gains $\gamma \;\in \;R^{K}$:

(3) $$Y_{\mathrm{fix}}\;=\;X\;B^{⊤}\;\mathrm{diag}\left(\gamma \right)\;\in \;R^{N\times K}.$$

the fixed frequency proxy bases are analytically defined rather than learned from data. Specifically, we use the first *K* one-dimensional DCT-II basis vectors constructed over the token feature dimension and order them from low to high frequency. We choose DCT because it is parameter-free, numerically stable, and provides an interpretable spectral decomposition that matches the frequency characteristics of image degradation in super-resolution. Specifically, the DCT basis is particularly effective at isolating periodic texture losses and global spectral inconsistencies, which are often difficult to explicitly characterize using purely spatial convolutions. In this way, each channel of $Y_{\mathrm{fix}}$ corresponds to a well-defined spectral mode, yielding lightweight yet interpretable routing cues. To move beyond the fixed subspace, we added a zero-initialized residual head $W_{\mathrm{res}}\;\in \;R^{d\times K}$:

(4) $$Y_{\mathrm{freq}}\;=\;Y_{\mathrm{fix}}\;+\;X\;W_{\mathrm{res}}\;\in \;R^{N\times K}.$$

This combination preserves interpretability (per-mode responses), while allowing task-specific deviations to emerge during training.

Spatial distortion branch (edge and noise proxies).

We project the tokens onto a scalar intensity with $w_{\mathrm{int}}\;\in \;R^{d}$ and reshape to an image:

(5) $$i\;=\;X\;w_{\mathrm{int}}\in R^{N\times 1}\;\overset{\mathrm{reshape}}{\to }\;I\in R^{B\times 1\times H\times W}.$$

Using Sobel kernels $S_{x},S_{y}$, we compute the gradients and edge magnitude (with a small ε):

(6) $$G_{x}=S_{x}*I,\;\;G_{y}=S_{y}*I,\;\;E=\sqrt{G_{x}^{2}+G_{y}^{2}+\varepsilon }\in R^{B\times 1\times H\times W}.$$

Let S=A∗I be a 3×3 mean filtering of *I*, and R=|I−S| be the high-frequency residual. We form a noise proxy by suppressing strong edges:

(7) N=ReLU(R−E).

These spatial proxies are designed to capture non-periodic degradations that are complementary to the frequency branch: the edge magnitude e identifies local structural blurring, while the noise proxy n detects high-frequency artifacts and irregular sensor noise. The flatten-token order and application of global learnable scales $\beta_{\mathrm{edge}},\beta_{\mathrm{noise}}$:

(8) $$e=\beta_{\mathrm{edge}}\cdot \mathrm{vec}\left(E\right),\;n=\beta_{\mathrm{noise}}\cdot \mathrm{vec}\left(N\right),\;e,n\in R^{N\times 1}.$$

Fusion and projection to gating space.

We concatenate the frequency vector with two scalars per token and project as follows:

(9) $$\begin{array}{cc} Z\;= & \mathrm{LN}\;\left(\;[\;Y_{\mathrm{freq}}\;;\;e\;;\;n\;]\;\right)\in R^{N\times (K+2)},\\ F\;= & \mathrm{Proj}\left(Z\right)\in R^{N\times D}. \end{array}$$

where Proj(·) is a linear projection parameterized by a weight matrix $W_{\mathrm{proj}}\in R^{(K+2)\times D}$. In the router (Section 3.3), we use the fused input $\tilde{X}=\left[\mathrm{LN}\left(X\right)\;∥\;\mathrm{LN}\left(F\right)W_{f}\right]$; thus, DAFE contributes but does not replace spatial features.

Fallback without (H,W).

If (H,W) is unknown, we approximate the edge or noise in the feature space as ${\hat{e}}_{i}=\frac{1}{d-1}\sum_{c=1}^{d-1}\left|x_{i,c+1}-x_{i,c}\right|,\;{\hat{n}}_{i}={\mathrm{Std}}_{c}\left(x_{i,:}\right)$, and reuse the same fusion.

Complexity and stability.

The dominant cost is one GEMM $XB^{⊤}$ of O(NdK), and the spatial branch adds three 3×3 single-channel convolutions, which is negligible compared with the backbone. We retain DAFE in FP16/BF16 but compute Sobel magnitude and layer norms in FP32 to avoid numerical issues. During warm-up, we optionally stop the gradients from the gate into the first DAFE block to stabilize the routing and then unfreeze them after a fixed number of steps.

### 3.3. DAEC: Distortion-Aware Expert Choice Routing

From distortion cues to capacity-aware routing. As shown in Figure 3 and detailed in Algorithm 1, DAEC transforms distortion-augmented inputs into capacity-controlled assignments. The experts claim tokens via Top-*C* selection, which yields predictable memory and latency and encourages role formation aligned with the degradation modes.

Affinity scores from fused inputs.

Given $\tilde{X}=\left[\mathrm{LN}\left(X\right)\;∥\;\mathrm{LN}\left(F\right)W_{f}\right]\in R^{N\times (d+d_{g})}$, a linear gate produces temperature-scale logits.

(10) $$A\;=\;\tilde{X}\;W_{g}/\tau ,\;a_{i,e}\;=\;{\left(A\right)}_{i,e},\;\;1\leq e\leq E.$$

Expert-choice with capacity and token fan-out.

We set the per-expert capacity to

(11) $$C\;=\;\left\lfloor \phi \cdot \frac{N}{E}\right\rfloor ,\;\phi \geq 1,$$

and each expert claims the Top-*C* tokens.

(12) $$S_{e}\;=\;\text{Top-}C\left({\left\{a_{i,e}\right\}}_{i=1}^{N}\right).$$

A token may be claimed by multiple experts, and after conflict resolution, we cap the per-token fan-out to ρ∈{1,2}:

(13) $$A\left(i\right)\;=\;\underset{e\in \{\;e|i\in S_{e}\;\}}{\text{Top-k}}\;a_{i,e}.$$

Tokens with A(i)=∅ take the lightweight fallback $F_{\mathrm{fb}}$ to guarantee coverage.

Dispatch, aggregation, and residual fusion.

Within the admitted set A(i), we compute the normalized weights

(14) $$w_{i,e}\;=\;\frac{\exp(a_{i,e})}{\sum_{e^{\prime }\in A\left(i\right)}\exp\left(a_{i,e^{\prime }}\right)}\;\left(e\in A\left(i\right)\right),$$

apply PS experts $E_{e}\left(\cdot \right)$ (Section 3.4), and aggregate:

(15) $$\begin{array}{cc} y_{i}\;=\; & \sum_{e\in A(i)}w_{i,e}\;E_{e}\left(x_{i}\right)\;+\;I\;\left[A(i)=\emptyset \right]\;F_{\mathrm{fb}}\left(x_{i}\right),\\ {\hat{x}}_{i}\;=\; & \mathrm{LN}(y_{i}+x_{i}). \end{array}$$

where $F_{\mathrm{fb}}$ is a lightweight MLP processing tokens not claimed by any expert to ensure complete coverage.

Stabilizers and objective.

We add three auxiliary terms to the task loss $L_{\mathrm{task}}$:

(16) $$\begin{array}{cc} {\bar{p}}_{e}\; & =\;\frac{1}{N}\sum_{i=1}^{N}\mathrm{softmax}{\left(a_{i,:}\right)}_{e}, \end{array}$$

(17) $$\begin{array}{cc} L_{\mathrm{imp}}\; & =\;\frac{E\cdot \sum_{e}{\bar{p}}_{e}^{2}-1}{E-1}\;(\mathrm{importance}\;\mathrm{smoothing}), \end{array}$$

(18) $$\begin{array}{cc} L_{\mathrm{cap}}\; & =\;\frac{1}{E}\sum_{e=1}^{E}\max\;\left(0,\;|S_{e}|\;-\;C\right)\;(\mathrm{overflow}\;\mathrm{penalty}), \end{array}$$

(19) $$\begin{array}{cc} L_{z}\; & =\;\frac{1}{N}\sum_{i=1}^{N}{\left(\log\sum_{e=1}^{E}\exp\left(a_{i,e}\right)\right)}^{2}\;(\mathrm{logit}\;\mathrm{range}). \end{array}$$

The full objective is $L=L_{\mathrm{task}}+\lambda_{1}L_{\mathrm{imp}}+\lambda_{2}L_{\mathrm{cap}}+\lambda_{3}L_{z}$.

Temperature schedule and utilization heuristics.

We annealed the routing sharpness to balance exploration and specialization: $\tau_{t}=\max\left(\tau_{\min},\tau_{0}\cdot \gamma^{\;t}\right)$. A global pre-fill heuristic reduces overflow by sorting pairs (i,e) by $a_{i,e}$ and greedily admitting them while respecting the capacity *C* and fan-out ρ.

Complexity and determinism.

The gating GEMM and subsequent Top-*C* selection have a combined complexity of $O\left(N(d+d_{g})E+E\cdot N\log C\right)$, where the second term arises from the batched partial sort. We retained the logits in FP32 to ensure numerical stability. We used a stable argsort and fixed seeds to ensure deterministic assignment across devices.
**Algorithm 1** DAEC (Expert-Choice with Capacity *C* and Fan-out ρ)  1:**Input:** tokens $X={\left\{x_{i}\right\}}_{i=1}^{N}$, DAFE features ${\left\{F_{i}\right\}}_{i=1}^{N}$, experts ${\left\{E_{e}\right\}}_{e=1}^{E}$, capacity factor ϕ, fan-out ρ  2:**Fuse** features: ${\tilde{x}}_{i}\;\leftarrow \;\left[\;\mathrm{LN}\left(x_{i}\right)\;∥\;\mathrm{LN}\left(F_{i}\right)W_{f}\;\right]$  3:**Gate** to get affinities: $a_{i,:}\;\leftarrow \;{\tilde{x}}_{i}W_{g}/\tau$  4:**Set** per-expert capacity: C←⌊ϕ·N/E⌋  5:**Experts claim** Top-*C* tokens: $S_{e}\;\leftarrow \;\text{Top-}C\left({\left\{a_{i,e}\right\}}_{i=1}^{N}\right)$ for e=1..E  6:**Resolve** conflicts (per-token fan-out): $A\left(i\right)\;\leftarrow \;\text{Top-k}\;\left\{a_{i,e}\;|\;i\in S_{e}\right\}$ with k=ρ  7:**Compute** weights for admitted experts: $w_{i,e}\;\leftarrow \;{\mathrm{softmax}}_{e\in A(i)}\left(a_{i,e}\right)$  8:**Aggregate** outputs:  9:**if** A(i)≠∅ **then**10:   $y_{i}\;\leftarrow \;\sum_{e\in A(i)}w_{i,e}E_{e}\left(x_{i}\right)$11:**else**12:   $y_{i}\;\leftarrow \;F_{\mathrm{fb}}\left(x_{i}\right)$ {Use fallback path}13:**end if**14:**Output** with residual connection: ${\hat{x}}_{i}\;\leftarrow \;\mathrm{LN}\left(y_{i}+x_{i}\right)$

### 3.4. PS-MoE: Parameter-Shared Experts with Band-Wise LoRA

Motivation and interface. As illustrated in Figure 4, the PS-MoE preserves the benefits of expert specialization while avoiding a parameter/VRAM explosion across B bands. Each expert shares a lightweight two-layer MLP trunk across the bands and attaches band-wise LoRA adapters to both projections. Routing is provided by DAEC (Section 3.3); this subsection focuses on the expert architecture.

Architecture.

We assume that $x\;\in \;R^{d}$ is a token and b∈{1,…,B} its band index (optionally encoded as a small embedding and concatenated to the gate input in Section 3.3). Expert $E_{e}$ shares weights $\left(W_{1},b_{1}\right)$ and $\left(W_{2},b_{2}\right)$ with GELU nonlinearity ϕ(·), plus per-band LoRA adapters of rank *r* and scaling $s=\alpha_{\;L}/r$:

(20) $$\begin{array}{cc} h_{\mathrm{shared}} & =\phi \;\left(xW_{1}+b_{1}\right),\\ h_{\mathrm{lora}} & =s\cdot \left(xA_{b}^{\left(1\right)}\right)B_{b}^{\left(1\right)},\\ h & =\phi \;\left(h_{\mathrm{shared}}+h_{\mathrm{lora}}\right),\\ y_{\mathrm{shared}} & =hW_{2}+b_{2},\\ y_{\mathrm{lora}} & =s\cdot \left(hA_{b}^{\left(2\right)}\right)B_{b}^{\left(2\right)},\\ E_{e}\;\left(x;b\right) & =y_{\mathrm{shared}}+y_{\mathrm{lora}}. \end{array}$$

Here, $A_{b}^{\left(l\right)}\in R^{d_{\mathrm{in}}\times r}$ and $B_{b}^{\left(l\right)}\in R^{r\times d_{\mathrm{out}}}$ denote the band-specific low-rank down-projection and up-projection matrices for layer *l*, respectively. We initialize all LoRA weights to zero so that experts start band-agnostic and specialize gradually.

Parameter and memory efficiency.

Parameters of one expert are as follows:

(21) $${\mathrm{Params}}_{\mathrm{per}\;\mathrm{expert}}\;=\;\underset{\mathrm{shared}\;\mathrm{trunk}}{\underset{︸}{2dh}}\;+\;\underset{\mathrm{LoRA}\;\mathrm{per}\;\mathrm{band}}{\underset{︸}{2\;B\;r\;(d+h)}}.$$

which is substantially smaller than B fully independent experts. At inference, the LoRA paths add only low-rank matvecs; the dominant cost remains in the shared trunk, keeping the latency predictable.

Placement and interaction with DAEC.

The PS-MoE replaces the dense FFN in Swin2SR-style blocks and is stacked across the depth. DAEC activates only a small subset of experts for each token; therefore, although the total capacity is large, the activated parameters per call remain modest. The shallow PS-MoE layers absorb coarse cross-band structures (guided by the global components of DAFE), whereas deeper layers refine band-dependent high frequencies.

Practical notes.

(i) We found that r∈{2,4,8} is effective; r=4 often offers the best accuracy–efficiency balance. (ii) Scaling: initializing $\alpha_{\;L}$ controls the relative strength of the adapters and is stable across datasets. (iii) For numerical robustness under AMP, we maintained the LayerNorms and LoRA accumulations in FP32. (iv) When ρ=2 in DAEC, experts tend to naturally split into “detail-recoverers” and “artifact-suppressors”; the band-wise adapters of PS-MoE prevent spectral ringing while preserving cross-band consistency.

## 4. Experiments

### 4.1. Tasks and Datasets

#### 4.1.1. Task

We evaluated single-image super-resolution (SISR) in two real-world multispectral settings: (i) SEN2VENµS at 2× and 4× upscaling, where Sentinel-2 LR inputs (10m and 20m) are mapped to VENµS HR references (5m); and (ii) OLI2MSI at 3× upscaling, where Landsat-8 OLI LR (30m) is mapped to Sentinel-2 MSI HR (10m). Unless otherwise noted, ablations were conducted in the SEN2VENµS 4× setting because of greater difficulty in fine-detail recovery. The metrics and efficiency follow Section 4.2 and Section 4.3.

#### 4.1.2. SEN2VENµS

SEN2VENµS is a large-scale paired cross-sensor dataset used for supervised RSISR. It provides co-registered Sentinel-2 LR patches at 10 m and 20 m ground sample distances (LR sizes 128×128 and 64×64, respectively), together with VENµS HR patches at 5 m (256×256). Pairs were acquired over the same location on the same day within a maximum time difference of 30 min, covering 29 regions over 2 years for a total of approximately 132,955 patch triplets. We followed the band protocol of the dataset: 2× used the S2 10 m bands {B2,B3,B4,B8} (blue, green, red, NIR), and 4× used the S2 20 m bands {B5,B6,B7,B8A} (red-edge and narrow-band NIR). The HR references were VENµS 5m for the corresponding scenes [38].

#### 4.1.3. OLI2MSI

The OLI2MSI dataset is a paired cross-sensor benchmark constructed by selecting relatively cloud-free image pairs over the same locations within a suitable temporal window. It couples Landsat-8 OLI LR patches at 30 m (160×160) with Sentinel-2 MSI HR patches at 10 m (480×480), thereby yielding a 3× upscaling factor [39]. The dataset contained 5325 pairs, split into 5225 for training and 100 for testing (we followed the official split). Following the original setting, we use three bands (RGB) as the input (L8: {B2,B3,B4}↔ S2: {B2,B3,B4}).

### 4.2. Implementation Details

#### 4.2.1. Hardware and Software

All experiments run on a single NVIDIA A800 80 GBGPU unless otherwise noted. We used PyTorch 2.1.0 with CUDA 12.1 and cuDNN 8.9 together with AMP (bfloat16), and LayerNorms and routing logits were accumulated in FP32 for numerical stability. Latency is reported as the synchronized wall-clock time per image (with torch.cuda.synchronize()), and VRAM is the peak-allocated memory via torch.cuda.max_memory_allocated(). We enabled deterministic cuDNN where feasible (with a slight throughput drop).

#### 4.2.2. Model Configuration

We adopt a Swin2SR-style backbone and replace dense FFNs with our DAEC + PS-MoE blocks. Routing uses Expert-Choice (EC) with a capacity factor ϕ=1.25 and a per-token fan-out ρ=2. Each expert is a two-layer MLP shared across bands, augmented with band-wise LoRA adapters (rank r=4, scaling $\alpha_{L}=8$) at both projections [19]; LoRA weights are zero-initialized so experts start band-agnostic. DAFE is attached before the gate at each MoE layer and provides lightweight distortion cues (frequency tokens and edge/noise proxies) that are concatenated with the spatial features for gating.

#### 4.2.3. Hyperparameter Selection

The default hyperparameters were determined through a stepwise ablation procedure under a fixed training budget rather than ad hoc tuning. Specifically, we first fixed the backbone, optimizer, learning-rate schedule, crop size, and batch setting, and then tuned the routing-related parameters and the parameter-efficient adaptation settings in a controlled manner. We started from a compact but stable Expert-Choice configuration and varied one factor at a time, including the number of experts *E*, the token fan-out ρ, the capacity factor ϕ, and the LoRA rank *r*, while keeping the remaining settings unchanged. All hyperparameter choices were made using the held-out validation subset from the training portion only, and the official test split was used strictly for the final evaluation. The final defaults (E=8, ρ=2, ϕ=1.25, and r=4) were selected because they consistently provided the best accuracy–efficiency trade-off, yielding strong PSNR/SSIM while maintaining stable routing, zero dropped tokens, and moderate memory overhead.

#### 4.2.4. Training Protocol (Common)

We use AdamW ($\beta_{1}=0.9$, $\beta_{2}=0.99$, weight decay $1\times 10^{-4}$). The learning rate increases linearly for five epochs to $6\times 10^{-4}$, followed by a cosine decay to $1\times 10^{-6}$. The gradients are clipped to a global norm of 1.0. Data augmentation: random horizontal/vertical flips and $90^{\circ }$ rotations. Unless otherwise specified, we use an effective batch of 192 via gradient accumulation (configs report micro-batch and accumulation). To stabilize early routing, we freeze gate ← DAFE gradients for the first 2 epochs and apply an exponential temperature schedule for the gate thereafter.

#### 4.2.5. Data Augmentation and Data Splits

We used random horizontal flips, vertical flips, and $90^{\circ }$ rotations because these transformations preserve the radiometric content and spatial semantics of remote-sensing scenes while improving orientation robustness and reducing overfitting. Since land-cover patterns and man-made structures may appear in arbitrary directions in satellite imagery, such geometric augmentation is a natural choice and does not alter the spectral correspondence between the LR and HR pairs. The augmentation was applied online during training, meaning that the stored dataset size did not physically increase; instead, the effective diversity of the training samples was enlarged through stochastic transformations across epochs. For dataset partitioning, we followed the official protocol and used 80% of the samples for training and the remaining 20% for testing. From the official training portion, we further held out a small subset for validation during hyperparameter selection, while the official test split was kept untouched for final reporting. Data augmentation was applied only to the training subset, whereas the validation and test subsets were kept unchanged for fair evaluation.

#### 4.2.6. Dataset-Specific Schedules (Consistent with Section 4.1)

##### SEN2VENµS (2×/4×)

The inputs follow the official S2 band groups [38] for 2×, {B2,B3,B4,B8} at 10 m LR crops of 128×128 with HR targets of 256×256 (5 m). For 4×, using {B5,B6,B7,B8A} at 20 m and cropping LR 64×64 with the HR targets of 256×256 (5 m). Training runs for 60 epochs; the other optimizer and routing settings are as described above.

##### OLI2MSI (3×)

The inputs are three bands (RGB) [39]. LR crops are 160×160 (30 m) with HR targets of 480×480 (10 m). Training was performed for 40 epochs using the same optimizer/schedule. Given the larger cross-sensor spectral shift, we enable a small-band embedding concatenated to the gate input; all other hyperparameters were kept identical for comparability.

#### 4.2.7. Sparse Efficiency Reporting

We report dynamic FLOPs as

$$\mathrm{FLOPs}={\mathrm{FLOPs}}_{\mathrm{MoE}\;\mathrm{trunk}}\cdot \frac{E[|A(i)|]}{E}+{\mathrm{FLOPs}}_{\mathrm{gate}}+{\mathrm{FLOPs}}_{\mathrm{DAFE}},$$

where, E[|A(i)|]≈ρ is the average number of admitted experts per token. The gate and DAFE overheads (∼2–3% by default) are included.

#### 4.2.8. Baselines and Fairness

To ensure a rigorous and equitable comparison, we re-trained several state-of-the-art Transformer-based restoration models—including SwinIR [1], Swin2SR [3], HAT [21], RGT [22], ESSAformer [23], FAT [40], and CGA [41]—on our dataset splits. All models were reproduced using a unified training protocol, encompassing identical budgets for epochs, optimizers, learning rate schedules, and data augmentations, without the use of test-time augmentation (TTA). Classical CNN-based benchmarks, such as EDSR [28], RCAN [29], SRMD [30], and ZSSR [31], are also included as representative references.

For efficiency benchmarking, all performance metrics—including latency, peak VRAM usage, and FLOPs—were measured under a standardized evaluation harness on an NVIDIA A800–80GB GPU. Measurements were conducted using automatic mixed precision (bfloat16) with a batch size of 1, targeting the native high-resolution (HR) patch dimensions for each dataset (256×256 for SEN2VENµS and 480×480 for OLI2MSI).

### 4.3. Metrics

#### 4.3.1. Evaluation Protocol

The restoration quality was evaluated using peak signal-to-noise ratio (PSNR) and structural similarity index measure (SSIM), both computed using the piq library [42,43,44,45,46,47]. Following standard SR practice, predictions and references were de-normalized to [0,1], and a 4-pixel boundary crop was applied uniformly across all methods and datasets. For multispectral data, the metrics were computed per band and per image and then averaged across bands and images, consistent with Equations (1) and (2). PSNR follows the standard closed-form definition (MAX=1), and SSIM uses the Gaussian window implementation from piq. Unless otherwise stated, the evaluations were performed without test-time augmentation and averaged over three fixed seeds.

#### 4.3.2. Efficiency Benchmarking

We measured latency as the synchronized wall-clock time per native high-resolution patch. (SEN2VENµS: 256×256; OLI2MSI: 480×480) on an A800–80GB GPU using AMP (bfloat16) and batch =1. VRAM corresponds to the peak-allocated memory during a single forward pass. Sparse FLOPs follow the formulation in Section 4.2, combining trunk FLOPs scaled by the expected number of admitted experts plus gating and DAFE overhead. In addition, we report the load-balance coefficient of variation to quantify expert utilization.

Complete formulas, aggregation rules, SSIM/PSNR configurations, alignment details, and reproducibility settings are provided in the Supplementary Materials.

### 4.4. Reproducibility and Statistical Reliability

All models were trained using three fixed random seeds, and we report the mean ± standard deviation across independent runs. Table 1 shows that the variation across runs is very small (e.g., std ≤ 0.04 dB across all datasets), indicating a stable training behavior. Because the number of repetitions is limited (n=3), we focused on consistent improvements rather than strong parametric significance claims. The performance gaps between the proposed method and the strongest baseline are consistent in all runs. Additional seeds and nonparametric significance analyses are presented in the Supplementary Material. All code, training configurations, and evaluation scripts will be released to support full reproducibility.

### 4.5. Comparison with State-of-the-Art

Table 1 reports the quantitative comparison with recent state-of-the-art methods on SEN2VENµS (2×, 4×) and OLI2MSI (3×). As presented in Table 1, our proposed method consistently outperforms all compared baselines across the three benchmark tasks. Notably, the recently introduced FAT [40] and CGA [41] demonstrate highly competitive performance; for instance, CGA achieves the second-best PSNR of 49.26 dB on SEN2VEN*μ*S 2×, while FAT reaches 45.81 dB on SEN2VEN*μ*S 4×. Despite these strong results from the latest state-of-the-art models, our DAEC + PS-MoE framework maintains a clear lead, surpassing the strongest competitors by 0.12 dB and 0.10 dB on the 2× and 4× tasks, respectively. More importantly, our method achieves these gains with significantly higher parameter efficiency. While CGA and FAT involve over 5.8 M parameters, our model utilizes only 4.59 M parameters—a reduction of approximately 20%—thereby demonstrating a superior trade-off between restoration fidelity and computational footprint.

### 4.6. Ablation Studies

#### 4.6.1. Are Cues Necessary? Effectiveness of DAFE

We treat frequency-like signals (e.g., DCT modes) as one branch of the cues within DAFE. Next, we examine the necessity of these cues.

As shown in Table 2, relying only on spatial features for routing yields a strong baseline (48.65 dB). However, adding simple learnable (MLP) or fixed (DCT) frequency proxies provides a significant improvement. Our full DAFE module, which combines fixed priors, learnable residuals, and spatial distortion proxies (edge/noise), achieves the best performance (49.38 dB), confirming that explicit multi-faceted distortion modeling is key to effective routing. This trend is consistent across datasets.

#### 4.6.2. Do Experts Divide Labor? DAEC Enables Specialization

Building on the above observation, we next ask whether better distortion cues merely improve routing accuracy, or whether they also induce a clearer division of labor among experts. To answer this, we perform a routing analysis at inference time. Specifically, we select 100 clean images and construct five input groups, including the original images, two levels of Gaussian blur, and two levels of Gaussian noise. The blur settings are defined as Blur-L with a 5×5 kernel and σ=1.2, and Blur-H with a 9×9 kernel and σ=2.4. The noise settings are defined as Noise-L with Gaussian noise level σ=15 and Noise-H with σ=35 (under the 0–255 image range). For each group, we record the routing decisions at every MoE layer and report the averaged Gini coefficient and Load CV^2^ as heatmaps, following the routing analysis in [13]. Here, the Gini coefficient measures the concentration of expert assignments, where a higher value indicates stronger specialization, while Load CV^2^ quantifies the imbalance of expert utilization, where a lower value indicates better load balance.

**Figure 5:**
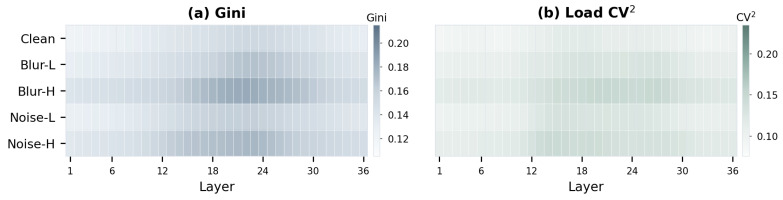
Layer-wise routing statistics under different distortion types and severities. (**a**) Gini coefficient of expert assignments. Higher values indicate stronger expert specialization. (**b**) Load CV^2^ across experts. Lower values indicate better load balance. From shallow to deep layers, stronger blur/noise distortions induce more concentrated expert selection, while the overall load imbalance remains controlled, showing that DAEC achieves specialization without sacrificing routing stability.

Figure 5 provides direct evidence that the experts do not behave uniformly across depth. In Figure 5a, the Gini coefficient generally increases from shallow to deeper layers, indicating that expert assignments become progressively more selective as restoration proceeds. This effect is particularly noticeable under stronger degradations such as Blur-H and Noise-H, where the deeper layers exhibit more concentrated expert usage. Such a trend suggests that severely degraded inputs rely more on specialized experts in later stages, when the model shifts from coarse restoration to more targeted detail repair.

Meanwhile, Figure 5b shows that although routing becomes more selective, the overall expert utilization remains stable across layers and distortion types. The load CV^2^ varies only moderately, implying that the observed specialization is not caused by pathological collapse to a few experts, but instead reflects an organized and controlled division of labor. Therefore, DAEC does not simply route tokens more aggressively; it enables depth-dependent expert specialization while preserving stable expert usage.

This qualitative observation is consistent with the quantitative results in Table 3. First, switching from traditional Token-Choice routing to Expert-Choice (EC) dramatically improves performance (e.g., +0.46 dB over Top-2). Second, augmenting the EC with our distortion-aware DAFE cues (DAEC) provides a substantial additional gain (+0.69 dB), pushing the PSNR to 49.38 dB, demonstrating that what cues are used for routing are as important as how routing is performed. Taken together, the figure and the table suggest that more informative distortion cues not only improve restoration quality, but also help the experts develop clearer functional specialization.

#### 4.6.3. Why Parameter-Shared MoE? Efficiency–Accuracy Trade-Off

Alternative expert parameterizations are compared using a unified budget.

In Table 4, we compare separate experts per band (i.e., 8×B experts in total), shared experts with no adapters, and shared experts with LoRA adapters of various ranks [19]. A fully shared model without adaptation already achieves a strong PSNR of 48.66 dB, outperforming the parameter-heavy per-band expert model (PSNR 48.18 dB), likely because the shared trunk effectively captures strong cross-band priors. Adding lightweight LoRA adapters further boosts the performance significantly; with rank 4 adapters, we achieve a PSNR of 49.38, which is +1.20 dB higher than the per-band expert baseline. Notably, this superior performance comes with a significant reduction in parameter count (4.59 M vs. 7.72 M) and lower VRAM. Rank 2 adapters are effective but slightly less so, whereas rank 8 shows a small performance drop compared with rank 4, indicating that a moderate adapter capacity is optimal. This confirms that parameter sharing combined with low-rank band-specific tuning is highly effective, yielding a favorable accuracy–efficiency trade-off (i.e., similar or better PSNR/SSIM at lower parameter, FLOPs, and VRAM cost).

Protocol overview. We evaluate the proposed DAFE→DAEC→PS-MoE pipeline using standard RSISR settings with a strong emphasis on fairness and reproducibility. Unless otherwise specified, all latency and VRAM numbers are measured on a single NVIDIA A800 80 GB using AMP (bfloat16), batch = 1, and routing uses expert choice with capacity factor ϕ and token fan-out ρ. We report dynamic sparse FLOPs that include both gating and DAFE overhead and compute PSNR and SSIM with the piq library under a documented, fixed evaluation protocol [44,45]. The baselines are retrained under identical budgets, and we report the mean ± std across multiple seeds [1,3,21,22,23,28,29,30,31].

#### 4.6.4. Additional Parameter Ablations

We conducted ablations on the SEN2VENµS 2× task (using four input bands) to validate each design choice. Unless otherwise stated, the full model configuration described above was used. Table 5 summarizes the results.

##### Fine-Grained Reading of Main Results

In smooth regions, DAEC avoids oversharpening by routing low-distortion tokens to conservative experts, leading to lower MAE while maintaining PSNR comparable to strong baselines. In texture-dominant areas, DAFE exposes frequency deficits that trigger expert specialization, yielding denser microtextures and a higher SSIM. Along strong edges, the scatter backfill preserves alignment, whereas the PS-MoE adapters prevent color ringing across bands.

##### Error-Type Comparison

Baseline methods either (i) introduce periodic ringing near edges when pushed to high-frequency recovery or (ii) smooth out high-frequency tiles to avoid artifacts [28,29]. Our cue extraction → expert specialization → efficiency-aware refinement pipeline reduces both failure modes by allocating tokens with distinct distortion patterns to different experts.

##### Layered Ablations (A1–A5)

A1: With vs. without DAFE (routing input only). Removing DAFE from the gate (while keeping experts unchanged) increases gate entropy and load skew in the early layers; PSNR drops notably in textured scenes. With DAFE, routing entropy decreases layer-wise, indicating clearer specialization. A2: Post-hoc concatenation vs. routing participation. Feeding *F* only to the backbone (not the gate) yields marginal gains; letting *F* participate in routing provides the bulk of improvements, confirming the “distortion→routing” causality. A3: Expert-Choice vs. Token-Choice; capacity *C*; with/without the load-balancing terms ($L_{\mathrm{imp}}$ and $L_{\mathrm{cap}}$). EC avoids token drop or overflow and improves stability [14,17]. Moderate capacities (covering ∼*ρ* active experts or tokens) perform best.

A4: PS-MoE vs. per-band vs. vanilla MoE. PS-MoE attains near-per-band accuracy at a fraction of the parameters and VRAM, and strictly dominates the vanilla MoE on the accuracy–efficiency curve when combined with cue-aware DAEC [15]. A5: Progressive removal of DAFE across depth. Disabling DAFE from deep to shallow layers reveals monotonic degradation, especially on edge and texture metrics, validating the “progressive repair” view.

##### Sensitivity to Number of Experts and Capacity

In Table 5, we vary the number of experts *E* and the capacity factor ϕ. With limited experts (e.g., E=4), the model lacks diversity and may drop tokens when ϕ=1.0. Increasing the number of experts to six or eight improves performance owing to more specialized experts, with diminishing returns beyond eight at this model size. Moderate overcapacity (ϕ=1.25) prevents overflow while keeping latency modest. Our default values (E=8, ρ=2, ϕ=1.25) achieve the best fidelity.

##### Hyperparameter Sensitivity

Among all tested hyperparameters, the model is most sensitive to the routing capacity and expert diversity, namely the number of experts *E*, the fan-out ρ, and the capacity factor ϕ. These parameters directly control whether tokens can be assigned to sufficiently specialized experts without causing overflow or expert under-utilization, and therefore have the largest impact on both restoration fidelity and routing stability. The LoRA rank *r* is the next most influential factor: increasing *r* from a very small value improves band-specific adaptation, but overly large ranks bring only marginal gains while increasing memory and computation. By contrast, once training is stable, the model is relatively less sensitive to minor changes in optimizer settings or warm-up details than to the routing-related design choices. This observation is consistent with our method formulation, where distortion-aware expert allocation is the central mechanism and parameter-efficient adaptation plays a secondary but still important role.

##### Qualitative Ablation Analysis

To complement the quantitative analysis, we present representative qualitative comparisons focusing on two critical components of the proposed framework: the distortion-aware cue extractor and the routing mechanism. Since the proposed model operates as a tightly coupled DAFE→DAEC→PS-MoE pipeline, the simultaneous removal of multiple components would compromise the validity of the comparison. Consequently, we adopt a function-oriented ablation strategy. Specifically, we evaluate two representative variants: (1) the exclusion of the DAFE module to assess the contribution of explicit distortion-aware cues; and (2) the substitution of the proposed DAEC with classical Top-1 token-choice routing to verify the efficacy of distortion-aware expert-choice assignment. These two variants are subsequently compared against the full model.

**Figure 6:**
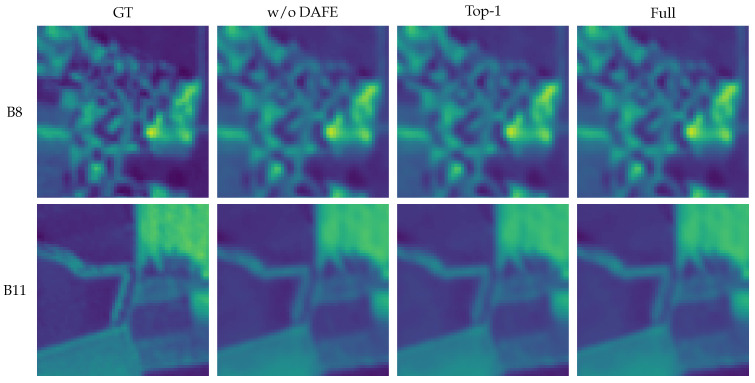
Qualitative ablation analysis on cropped regions from bands B8 and B11. For each row, regions are cropped using identical coordinates across all methods. The exclusion of DAFE results in degraded recovery of local structures, whereas replacing the proposed distortion-aware expert-choice routing with classical Top-1 token-choice routing leads to instability in challenging regions. In contrast, the full model yields sharper boundaries and higher fidelity in local structures, consistent with the quantitative improvements reported in the ablation study.

As illustrated in Figure 6, visual differences are subtle yet consistent across the representative bands. In band B8, the absence of DAFE results in inferior local structure recovery, demonstrating that explicit distortion-aware cues assist the router in distinguishing degradation patterns. When the DAEC is replaced by Top-1 token-choice routing, restoration stability decreases in complex regions, indicating the necessity of distortion-aware expert-choice assignment for handling heterogeneous spatial degradations. We observe a similar trend in band B11, where the full model generates structures visually closer to the GT. Overall, these qualitative observations align with the quantitative gains reported in the ablation study.

### 4.7. Qualitative Visual Comparisons

**Figure 7:**
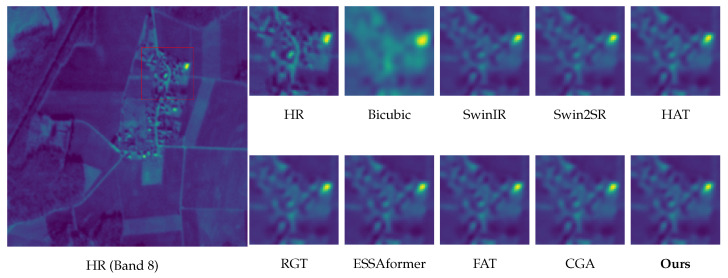
Visual comparison of 4× SISR on Sen2Ven*μ*s Band 8 (NIR). Our method maintains sharp corner geometry without the ringing artifacts observed in HAT and SwinIR.

**Figure 8:**
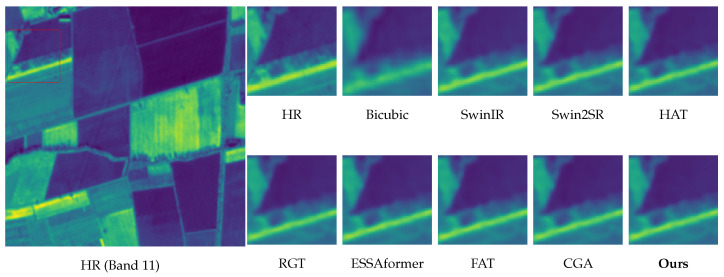
Visual comparison of 4× SISR on Sen2Ven*μ*s Band 11 (SWIR). Our method successfully recovers natural granular details compared to ESSAformer and RGT.

Visual Analysis. We focus our qualitative evaluation on the challenging 4× super-resolution regime because it presents a rigorous test for detailed recovery and artifact suppression. Figure 7 and Figure 8 show the results for band 8 (NIR) and band 11 (SWIR), respectively. These bands were selected to represent distinct restoration paradigms: band 8 features high-contrast geometric boundaries (testing edge stability), whereas band 11 contains dense, complex ground details (testing microtexture synthesis). Additional results for the other bands and the 2× scale are provided in the Supplementary Material. These three key observations support our quantitative gains.

**Figure 9:**
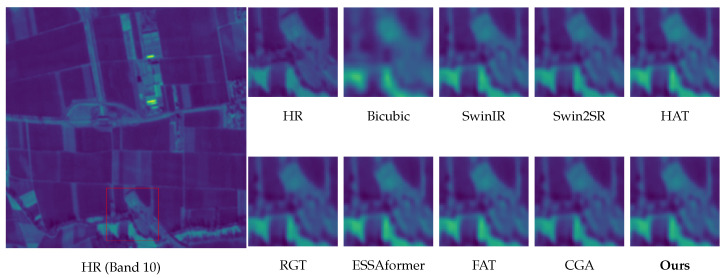
Visual comparison of 4× SISR on Sen2Ven*μ*s Band 10 (Cirrus). Our method preserves distinct cloud-land boundaries without the excessive oversmoothing found in ESSAformer and HAT.

**Figure 10:**
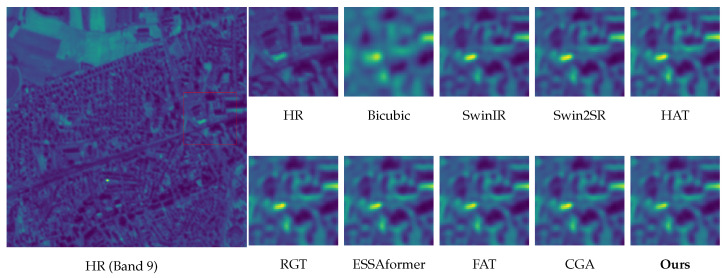
Visual comparison of 4× SISR on Sen2Ven*μ*s Band 9 (Water Vapour). Our method restores structural atmospheric gradients without the severe blurring or aliasing artifacts observed in Swin2SR and RGT.

1. Edge fidelity without ringing (Band 8). As shown in Figure 7, baselines, such as HAT and SwinIR, tend to oversharpen high-contrast edges, introducing halo-like overshoot or ringing artifacts. In contrast, our method—guided by the explicit edge proxies of DAEC—preserves sharp, continuous boundaries while suppressing Gibbs phenomena.
2. Robustness to atmospheric gradients and boundaries (Bands 9 & 10). Bands 9 (Water Vapour) and 10 (Cirrus) often contain diffuse atmospheric gradients and delicate cloud-land interfaces that are prone to distortions. As illustrated in Figure 9 and Figure 10, while RGT and Swin2SR exhibit severe aliasing or blurring in these low-contrast regions, our model accurately restores structural gradients and sharpens cloud boundaries. This demonstrates that our frequency-aware DAFE effectively handles spectral-specific textural characteristics without collapsing into oversmoothed results.
3. Texture recovery without hallucination (Band 11). For texture-intensive regions (Figure 8), Transformer-based methods often diverge: ESSAformer tends to over-smooth fine granules, while RGT risks generating artificial grid-like patterns. Our model leverages the frequency cues of DAFE to reconstruct natural micro-textures that align with the HR reference, avoiding both blurriness and generative hallucination.
4. Structural consistency at scale. At 4× upscaling, per-band inconsistencies often manifest as checkerboard artifacts or spectral aliasing (seen in Swin2SR). Our PS-MoE design, which shares a common expert trunk across bands, enforces strong structural consistency, ensuring that restored details remain geometrically aligned across the spectral stack.

## 5. Discussion

The results indicate that the performance gains of the proposed method stem not primarily from scaling the model size, but from aligning sparse computation with the degradation characteristics of multispectral RSISR. In contrast to conventional Transformer-based restoration models that process all tokens uniformly, our framework incorporates explicit distortion cues into the routing mechanism, thereby enabling the allocation of different regions to the most suitable experts. This capability is particularly critical for multispectral imagery, in which degradation severity and frequency loss typically vary across spatial locations and spectral bands.

In the context of related work, the proposed framework functions as a bridge between two distinct research paradigms: robust restoration backbones and sparse conditional computation. While prior restoration models facilitate powerful feature extraction and generic MoE methods enhance efficiency through token-dependent expert selection, our results demonstrate that routing in multispectral super-resolution is significantly more effective when guided by degradation-aware cues rather than generic feature similarity alone. This observation explains the superior trade-off between accuracy and efficiency achieved by the proposed method compared to recent baselines.

Despite these contributions, the proposed method exhibits certain limitations. First, the routing capacity is governed by a fixed global capacity factor, which may prove suboptimal for scenes with highly uneven degradation. Second, the framework does not explicitly model inter-band misregistration or sensor-dependent imaging discrepancies. Third, since the experiments are conducted primarily on supervised benchmarks with limited sensor diversity, the generalization of the proposed routing strategy to complex real-world scenarios necessitates further validation. Addressing these limitations constitutes a key direction for future research.

## 6. Conclusions

In this study, we reframe multispectral super-resolution (RSISR) as a sequence of cue extraction, expert specialization, and adaptive repair. By instantiating this view with explicit distortion tokenization (DAFE), capacity-constrained routing (DAEC), and parameter-shared experts (PS-MoE), we transform the frequency priors from passive features into active routing signals. Extensive evaluations of SEN2VEN*μ*S and OLI2MSI demonstrate that this paradigm establishes new state-of-the-art performance, matching or surpassing heavy Transformer baselines while significantly reducing FLOPs and memory overhead under realistic deployment budgets. Qualitative and quantitative analyses were used to validate the design choices. DAFE successfully localizes high-frequency deficits and noise, enabling the router to assign texture-dominated and structure-dominated regions to specialized experts without introducing artifacts. Furthermore, our PS-MoE architecture effectively reconciles the tension between cross-band structural consistency and band-specific statistics, offering a scalable solution for multispectral imagery. Ablation studies have confirmed that each component, from explicit cue extraction to low-rank adaptation, is essential for the observed accuracy and efficiency gains. Although effective, our method currently relies on a fixed global capacity and assumes reliable co-registration. Future work will explore adaptive capacity allocation to densify computation only where required and incorporate physics-based priors (e.g., MTF models) to better handle sensor misalignment or unsupervised regimes. We hope that this study encourages further research that treats distortion not only as a feature to be processed but also as a signal to guide how repair is performed.

## Figures and Tables

**Table 1:** Quantitative comparison with state-of-the-art on SEN2VENµS (2×, 4×) and OLI2MSI (3×). Best results in **bold**. Values are mean ± std over 3 seeds.

Method	Params (M)	FLOPs (G)	PSNR (dB)↑	SSIM↑	Latency (s)↓	VRAM (MiB)↓
SEN2VENµS 2× (Sentinel-2 → VENµS, 4-band input; HR 256×256)						
Bicubic	—	—	45.55 ± 0.00	0.9883 ± 0.0000	—	—
SwinIR [1]	4.68	171.0	48.69 ± 0.02	0.9932 ± 0.0002	0.232	2600
Swin2SR [3]	5.33	168.0	48.86 ± 0.03	0.9937 ± 0.0003	0.225	2550
HAT [21]	5.06	220.0	48.98 ± 0.04	0.9950 ± 0.0001	0.278	3400
RGT [22]	5.11	185.0	49.05 ± 0.02	0.9940 ± 0.0003	0.248	2900
ESSAformer [23]	4.45	175.0	49.20 ± 0.03	0.9953 ± 0.0002	0.238	2800
FAT [40]	5.81	194.4	49.01 ± 0.01	0.9949 ± 0.0002	0.268	3600
CGA [41]	5.95	185.0	49.26 ± 0.04	0.9953 ± 0.0001	0.271	3400
**Ours (DAEC + PS-MoE)**	**4.59**	**136.9**	**49.38 ± 0.02**	**0.9953 ± 0.0002**	**0.207**	**2600**
SEN2VENµS 4× (Sentinel-2 → VENµS, 4-band input; HR 256×256)						
Bicubic	—	—	42.04 ± 0.00	0.9764 ± 0.0000	—	—
SwinIR [1]	4.68	173.5	45.34 ± 0.04	0.9825 ± 0.0003	0.236	2620
Swin2SR [3]	5.33	170.4	45.47 ± 0.02	0.9828 ± 0.0004	0.228	2570
HAT [21]	5.06	223.1	45.53 ± 0.03	0.9831 ± 0.0002	0.282	3440
RGT [22]	5.11	187.2	45.47 ± 0.04	0.9828 ± 0.0003	0.252	2920
ESSAformer [23]	4.45	177.3	45.80 ± 0.02	0.9839 ± 0.0002	0.241	2720
FAT [40]	5.81	192.1	45.81 ± 0.03	0.9836 ± 0.0002	0.271	3640
CGA [41]	5.95	189.0	45.56 ± 0.01	0.9833 ± 0.0001	0.290	3280
**Ours (DAEC + PS-MoE)**	**4.59**	**139.8**	**45.91 ± 0.03**	**0.9847 ± 0.0003**	**0.214**	**2680**
OLI2MSI 3× (Landsat-8 OLI → Sentinel-2 MSI, 3-band input; HR 480×480)						
Bicubic	—	—	42.18 ± 0.00	0.9768 ± 0.0000	—	—
SwinIR [1]	4.68	171.0	44.60 ± 0.02	0.9820 ± 0.0004	0.260	2400
Swin2SR [3]	5.33	168.0	45.85 ± 0.03	0.9874 ± 0.0003	0.252	2380
HAT [21]	5.06	220.0	44.95 ± 0.02	0.9862 ± 0.0002	0.297	3200
RGT [22]	5.11	185.0	45.61 ± 0.04	0.9816 ± 0.0003	0.268	2850
ESSAformer [23]	4.45	175.0	44.92 ± 0.03	0.9801 ± 0.0004	0.258	2450
FAT [40]	5.81	194.1	45.61 ± 0.01	0.9812 ± 0.0002	0.298	3680
CGA [41]	5.95	181.0	45.69 ± 0.03	0.9893 ± 0.0001	0.288	3270
**Ours (DAEC + PS-MoE)**	**4.59**	**137.2**	**45.94 ± 0.02**	**0.9911 ± 0.0002**	**0.242**	**2520**

Note. All results were measured using an NVIDIA A800–80GB with AMP (bfloat16), batch =1, and native HR patch sizes (see Table caption). PSNR/SSIM was computed using piq [44,45] with per-band→per-image averaging and a 4-pixel border crop; no TTA. Sparse FLOPs include gate+DAFE overhead, and routing uses expert choice with a capacity factor ϕ and fan out ρ.

**Table 2:** Effect of cue source on SEN2VENµS 2×. Values are mean ± std over 3 seeds.

Routing Cues	Extra Params (M)	Extra FLOPs (G)	PSNR (dB)↑	SSIM↑	Note
Spatial-only (baseline)	0	0	48.65 ± 0.04	0.9918 ± 0.0003	No frequency cues (spatial tokens only)
+MLP	+0.03	+0.3	48.98 ± 0.03	0.9937 ± 0.0002	Shallow learnable frequency proxy
+Fixed DCT	+0.05	+0.3	49.03 ± 0.02	0.9942 ± 0.0003	Handcrafted global (fixed DCT modes)
+DAFE (Ours)	+0.24	+3.5	49.38 ± 0.03	0.9953 ± 0.0002	Learnable multi-branch (DCT+residual+edge/noise)

Note. Same protocol as Table 1.

**Table 3:** Routing mechanism ablation on SEN2VENµS 2×. Values are mean ± std over 3 seeds.

Routing Configuration	Experts *E*	Router Type	Features	ϕ	PSNR (dB)↑	SSIM↑	Latency (s)↓
Token-Choice (Top-1)	8	Token-Choice	No	1.25	47.95 ± 0.04	0.9850 ± 0.0004	0.202
Token-Choice (Top-2)	8	Token-Choice	No	1.25	48.23 ± 0.03	0.9895 ± 0.0003	0.204
Expert-Choice (EC)	8	Expert-Choice	No	1.25	48.69 ± 0.02	0.9933 ± 0.0002	0.206
DAEC (Ours)	8	Expert-Choice	Yes	1.25	49.38 ± 0.03	0.9953 ± 0.0003	0.207

Note. EC uses ρ=2 unless specified.

**Table 4:** Ablation on expert parameterization on SEN2VENµS 2×. PSNR values are mean ± std over 3 seeds.

Expert Parameterization	Experts	Adapter Type	Rank *r*	Params (M)	VRAM (MiB)	FLOPs (G)	PSNR (dB)↑	APC (K)/SPC (K)
Per-band experts	8×B	–	–	7.72	3100	138.3	48.18 ± 0.04	34,120/262,810
Shared experts (no adaptation)	8	–	–	4.15	2200	136.4	48.66 ± 0.03	33,480/131,760
Shared + LoRA adapters	8	LoRA	2	4.34	2260	136.6	48.83 ± 0.02	33,490/131,770
Shared + LoRA adapters (Ours)	8	LoRA	4	4.59	2340	136.9	49.38 ± 0.03	33,499/131,779
Shared + LoRA adapters	8	LoRA	8	5.07	2480	137.5	49.30 ± 0.04	33,520/131,800

Note. All results were measured using an NVIDIA A800–80GB with AMP (bfloat16), batch =1, and native HR patch sizes (see Table caption). PSNR/SSIM was computed using piq [44,45] with per-band→per-image averaging and a 4-pixel border crop; no TTA. Sparse FLOPs include gate+DAFE overhead, and routing uses expert choice with a capacity factor ϕ and fan out ρ.

**Table 5:** Model scaling ablation on SEN2VENµS 2×: Values are mean ± std over 3 seeds.

*E*	Fan-Out ρ	ϕ	PSNR (dB)↑	SSIM↑	Latency (s)↓	Dropped Tokens (%)↓	Notes
4	1	1.00	48.36 ± 0.02	0.9921 ± 0.0003	0.198	0.2%	Insufficient capacity (some drops)
6	1	1.25	48.95 ± 0.03	0.9937 ± 0.0002	0.204	0.0%	Good trade-off
8	2	1.25	49.38 ± 0.02	0.9953 ± 0.0003	0.207	0.0%	Default (max quality)

Note. EC fan-out ρ shown per row.

## Data Availability

The datasets analyzed in this study are publicly available. The SEN2VENµS dataset is available at its official repository, and the OLI2MSI dataset can be obtained from the corresponding benchmark release. No new datasets were generated during the current study.

## Supplementary Materials

The following supporting information can be downloaded at: https://www.mdpi.com/article/10.3390/s26072186/s1, Document S1: Detailed experimental configurations, formulas, and reproducibility settings; Figure S1: Additional qualitative visual comparisons for other spectral bands and the 2× upscaling task; Figure S2: Comparison of 2× SISR on Sen2Venμs, single-band view: B9 (Venμs 5 m); Figure S3: Comparison of 2× SISR on Sen2Venμs, single-band view: B10 (Venμs 5 m); Figure S4: Comparison of 2× SISR on Sen2Venμs, single-band view: B11 (Venμs 5 m); Figure S5: Comparison of 4× SISR on Sen2Venμs, single-band view: B8 (red edge, Venμs 5 m); Figure S6: Comparison of 4× SISR on Sen2Venμs, single-band view: B9 (Venμs 5 m).

## Abbreviations

The following abbreviations are used in this manuscript:

DAFE	Distortion-Aware Feature Extractor
DAEC	Distortion-Aware Expert Choice
PS-MoE	Parameter-Shared Mixture-of-Experts
MoE	Mixture-of-Experts
LoRA	Low-Rank Adaptation
RSISR	Remote Sensing Image Super-Resolution
SR	Super-Resolution
PSNR	Peak Signal-to-Noise Ratio
SSIM	Structural Similarity Index Measure
FLOPs	Floating Point Operations
VRAM	Video Random Access Memory
DCT	Discrete Cosine Transform
FFN	Feed-Forward Network
EC	Expert-Choice
PIQ	Perceptual Image Quality
SEN2VEN*μ*S	Sentinel-2 to Venus
OLI2MSI	Operational Land Imager to MultiSpectral Instrument
