# Supplementary material for: Distortion-Aware Routing and Parameter-Shared MoE for Multispectral Remote Sensing Super-Resolution
Source: Sensors (Basel). 2026 Apr 1;26(7):2186. doi: 10.3390/s26072186 (PMC13075315; doi:10.3390/s26072186)
Supplement: Supplementary file 1 [file sensors-26-02186-s001.zip › sensors-4160616-supplementary.pdf]

sensors

MDPI

Article

Supplementary Material for: Distortion–Aware Routing and Parameter–Shared MoE for Multispectral Remote Sensing Super-Resolution

Shuo Yang[1,2], Shi Chen[1,*], Yuxuan Liu[1,2,†] and Tianhui Zhang[1,2,†]

1 National Space Science Centre, Chinese Academy of Sciences, Beijing 100190, China; yangshuo23@mails.ucas.ac.cn; chenshi@nssc.ac.cn; liuyuxuan231@mails.ucas.ac.cn; zhangtianhui22@mails.ucas.ac.cn
2 University of Chinese Academy of Sciences, Beijing 100049, China
* Correspondence: chenshi@nssc.ac.cn (S.C.)
† † These authors contributed equally to this work.

Metrics (Full Details)

Quality Metrics

We compute PSNR and SSIM using the `piq` library [1,2]. Model outputs and references are de-normalized to $[0, 1]$ and cropped with a 4-pixel boundary to avoid upsampling artifacts. All metric computations are performed in FP32 for numerical stability.

Per-band Aggregation

For restored and reference stacks $\hat{Y}, Y \in \mathbb{R}^{B\times\mathcal{B}\times H\times W}$, PSNR and SSIM are computed per-band, per-image, then averaged:

$$\mathrm{PSNR} = \frac{1}{B\mathcal{B}} \sum_{b=1}^{B} \sum_{c=1}^{\mathcal{B}} \mathrm{PSNR}_{\texttt{PIQ}}(\hat{Y}_{b,c}, Y_{b,c}),$$

and similarly for SSIM. Each band is treated independently.

PSNR Definition

For $X, Y \in [0, 1]$,

$$\mathrm{PSNR} = 10 \log_{10}\left(\frac{1}{\mathrm{MSE}}\right), \quad \mathrm{MSE} = \frac{1}{HW} \sum_{ij} (X_{ij} - Y_{ij})^2.$$

`piq` implements the closed-form with an internal ε.

SSIM Settings

SSIM uses a Gaussian window with `piq` defaults (kernel size, σ). We evaluate each spectral band independently; no luminance conversion is applied.

Border Handling and Alignment

A 4-pixel crop is used for all datasets/methods. If a model outputs different spatial support, we center-crop to the common region. All datasets provide co-registered paired HR/LR inputs.

Check for updates

Academic Editor: Arturo Sanchez-Azofeifa

Received: 2 February 2026
Revised: 9 March 2026
Accepted: 29 March 2026
Published: 1 April 2026

Copyright: © 2026 by the authors. Licensee MDPI, Basel, Switzerland. This article is an open access article distributed under the terms and conditions of the Creative Commons Attribution (CC BY) license.

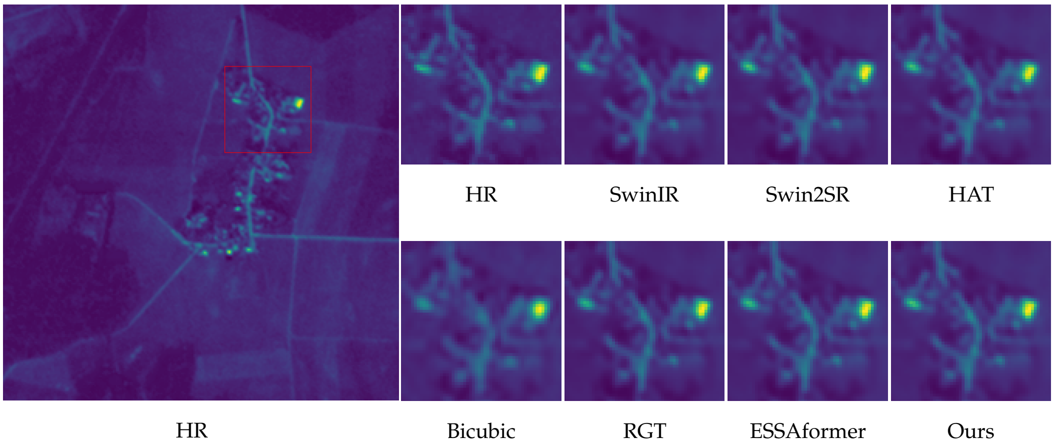

Figure S1. Comparison of **2**× SISR on **Sen2Venµs, single-band** view: **B8 (red edge, Venµs 5 m)**. Left: HR (B8) with a red ROI box. Right: zoomed ROI crops; the **top-left tile is the HR crop (HR)**. Remaining tiles (left→right, top then bottom): SwinIR, Swin2SR, HAT, Bicubic, RGT, ESSAformer, **Ours**.

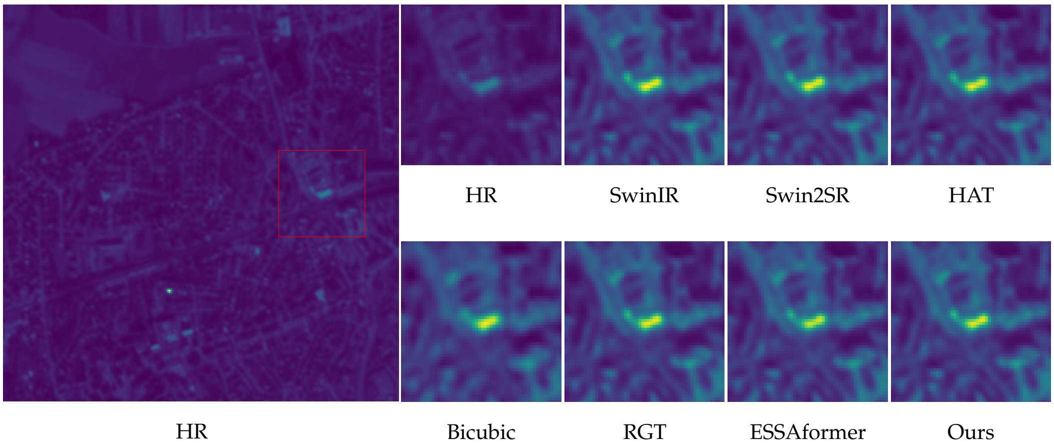

Figure S2. Comparison of **2**× SISR on **Sen2Venµs, single-band** view: **B9 (Venµs 5 m)**.

Efficiency Metrics

Latency is measured as synchronized wall-clock time per HR patch (SEN2VENµS: 256×256, OLI2MSI: 480×480) on A800–80GB with AMP (bfloat16) and batch$=1$. VRAM is peak allocated memory. Sparse FLOPs follow:

$$\text{FLOPs} = \text{FLOPs}_{\text{MoE trunk}} \cdot \frac{\mathbb{E}[|\mathcal{A}(i)|]}{E} + \text{FLOPs}_{\text{gate}} + \text{FLOPs}_{\text{DAFE}}.$$

We also report load-balance variation CV^2 across experts.

Reproducibility

All metrics are averaged over 3 seeds. We verified that `piq` reproduces closed-form PSNR on synthetic data. Flip/rotate TTA yields marginal changes (< 0.05 dB), so we disable TTA by default.

Qualitative Visual Comparisons

Reading guide for qualitative figures.

Each qualitative figure is a *single-band* comparison on Sen2Venµs: **B8**, **B9**, **B10**, or **B11** (Venµs HR at 5 m). The *left* panel shows the Venµs HR reference for that band with a single red ROI box. The *right* panel is a 2×4 grid of 256×256 crops: the **top-left tile is the HR crop**

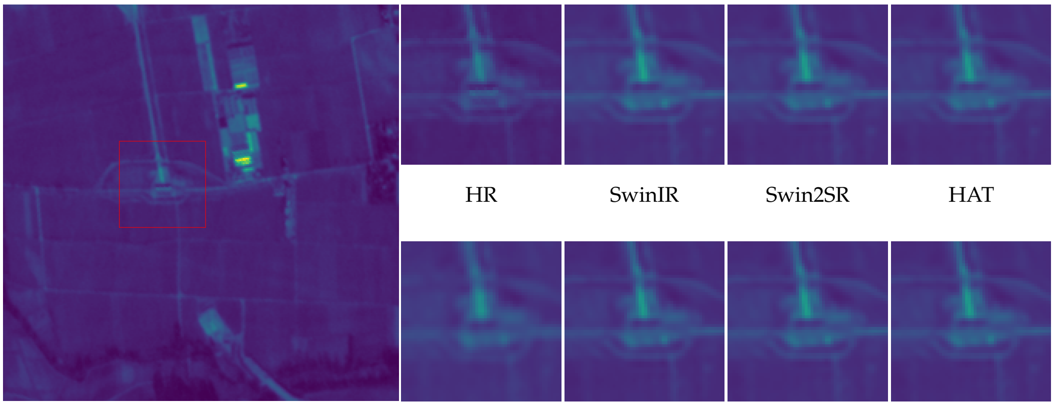

Figure S3. Comparison of **2**× SISR on **Sen2Venµs, single-band** view: **B10 (Venµs 5 m)**.

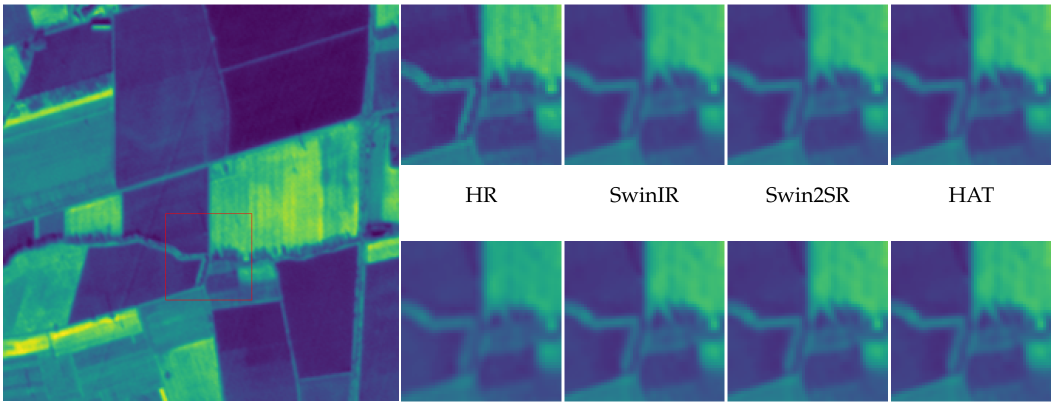

Figure S4. Comparison of **2**× SISR on **Sen2Venµs, single-band** view: **B11 (Venµs 5 m)**.

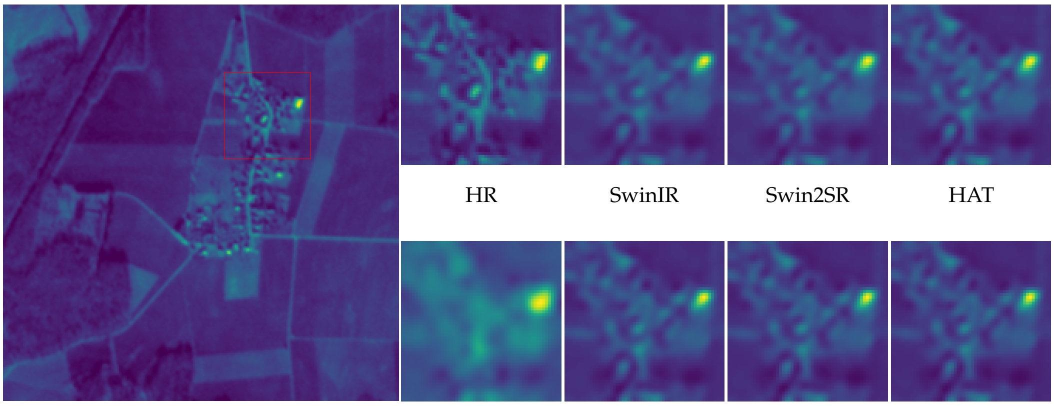

Figure S5. Comparison of **4**× SISR on **Sen2Venµs, single-band** view: **B8 (red edge, Venµs 5 m)**.

extracted from the red box and labeled **HR**. The remaining seven tiles appear in a fixed order (left → right, top then bottom): **SwinIR**, **Swin2SR**, **HAT**, **Bicubic**, **RGT**, **ESSAformer**, **Ours**. All methods use the identical ROI; no TTA; a 4-pixel border is cropped for fairness.

Analysis (B8, 2×).

Edge continuity. Even at 2×, Bicubic blurs boundaries; HAT/Swin2SR sharpen well but leave mild halos. **Ours** keeps crisp edges with less glow and improved corner geometry. *Micro-texture.* **Ours** enhances canopy/roof granularity without noise inflation; RGT

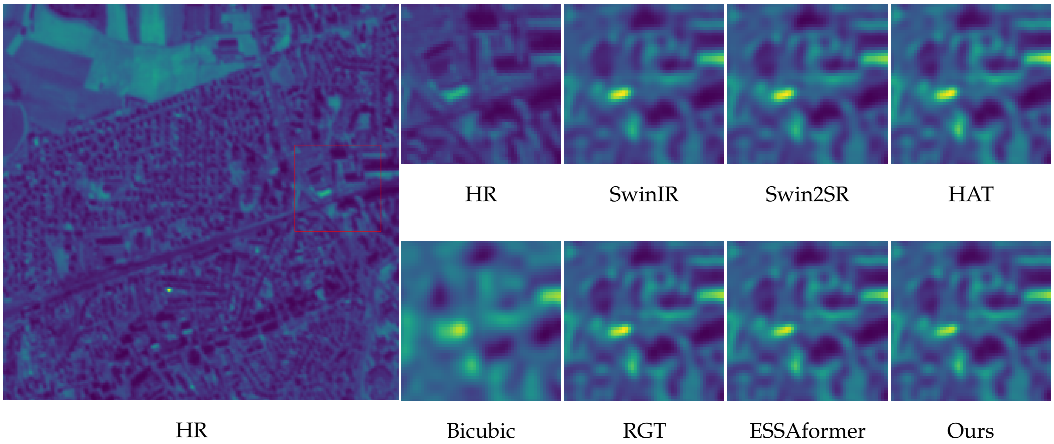

Figure S6. Comparison of **4**× SISR on **Sen2Venμs**, **single-band** view: **B9 (Venμs 5 m)**.

sometimes exaggerates periodicity; ESSAformer trends smooth. *Artifacts.* Ringing is rarer at 2×, yet **Ours** still shows the cleanest corners among sharp baselines.

Analysis (B9, 2×).

Thin structures. **Ours** preserves slim edges with fewer breaks than Bicubic/ESSAformer and less halo than HAT/SwinIR. *Texture realism.* Relative to RGT's repetitive patterns, **Ours** produces realistic micro-texture aligned with HR detail density. *Artifacts.* Faint aliasing present in Swin2SR/HAT is largely absent in **Ours**.

Analysis (B10, 2×).

Structure and corners. **Ours** retains corner sharpness without overshoot; Swin2SR/HAT are sharp but may halo at bright–dark corners. *Textures.* Compared to ESSAformer's smoothness and RGT's periodicity bias, **Ours** delivers detailed yet stable textures closer to HR.

Analysis (B11, 2×).

Edges. **Ours** provides the most continuous boundaries among sharp baselines, with fewer halos. *Micro-texture.* Detail is enhanced without false high-frequency bursts; RGT sometimes induces weak grid regularity; ESSAformer remains smooth. *Artifacts.* Practically no checkerboard or ringing is observed with **Ours**.

Analysis (B8, 4×).

Edge fidelity. In the HR crop (top-left of the right grid), strong boundaries are crisp; Bicubic blurs these, while SwinIR/Swin2SR and HAT sharpen but produce faint halos at bright–dark edges. **Ours** preserves edge sharpness with minimal over-shoot, improving line continuity along long man-made edges. *Fine texture.* ESSAformer remains conservative (smoothed canopy/roof granularity). RGT sometimes imprints weak periodic patterns on repetitive structures. **Ours** restores denser micro-texture without speckle, following the reference's local contrast. *Artifacts.* Checkerboard/ringing visible on sharp corners for Swin2SR/HAT under 4× are largely reduced by **Ours**, yielding cleaner junctions.

Analysis (B9, 4×).

Thin structures. Bicubic/ESSAformer under-resolve narrow features; HAT/Swin2SR sharpen them yet often leave halo glow. **Ours** retains thin lines with fewer breaks and lower halo intensity. *Texture vs. noise.* RGT tends to replicate micro-patterns; SwinIR can over-accentuate small periodicity. **Ours** keeps balanced texture with restrained HF boosting,

https://doi.org/10.3390/s26072186

avoiding granular noise. *Artifacts.* Mild aliasing/ringing around tiny corners evident in Swin2SR/HAT is suppressed by **Ours**.

Analysis (B10, 4×).

Structural accuracy. Bicubic over-smooths; ESSAformer misses sub-structures. SwinIR/HAT intensify edges but show slight overshoot on high-contrast corners. **Ours** keeps edges crisp with reduced overshoot and better corner geometry. *Micro-texture robustness.* **Ours** retrieves fine surface variations without band-dependent grain; RGT occasionally leaves grid-like regularity on repetitive textures. *Artifact control.* Checkerboard/aliasing are noticeably lower with **Ours** in the highest-frequency patches.

Analysis (B11, 4×).

Edges. **Ours** keeps boundary lines continuous with less halo than HAT/Swin2SR and much sharper contours than Bicubic/ESSAformer. *Textures.* Compared with conservative smoothing (ESSAformer) and periodicity bias (RGT), **Ours** yields detailed yet natural textures closer to the HR reference. *Artifacts.* Ringing is minimal and no checkerboard is visible on fine corners, indicating effective distortion-aware routing at large scale.

References

1. Kastryulin, S.; Zakirov, J.; Prokopenko, D.; Dylov, D.V. Pytorch image quality: Metrics for image quality assessment. *arXiv preprint arXiv:2208.14818* **2022**.
2. Team, P. PyTorch Image Quality (PIQ). https://github.com/photosynthesis-team/piq, 2020. Software.

https://doi.org/10.3390/s26072186
